# NE‐58025: A bisphosphonate with a low hydroxyapatite binding affinity

**DOI:** 10.1002/jbm4.10502

**Published:** 2021-05-07

**Authors:** Michael Pazianas

**Affiliations:** ^1^ Institute of Musculoskeletal Sciences Oxford University Oxford UK

## Peer review

The peer review history for this article is available at https://publons.com/publon/10.1002/jbm4.10502.

To the Editor:

I read with interest the article by Coffman et al.^(^ ^1^ ^)^ on the reversible, low hydroxyapatite (HAP) binding affinity and high farnesyl pyrophosphate synthase (FPPS) inhibition, bisphosphonate (BP) compound NE‐58024. The authors suggest that NE‐58025 may have clinical utility as an anti‐resorptive agent. However, they did not address potential drawbacks.

As the authors correctly state, moderate to high affinity to bone allows BPs to selectively target and adhere strongly to bone mineral in particular at sites of highest bone turnover, and then being internalized by osteoclasts during the resorption process. Low HAP binding affinity, although highly desirable, could significantly reduce the local availability unless the compound with such attributes is administered at pharmacological doses. Hence, among other systemic effects, there is a considerable risk that non‐osteoclast cells capable of internalizing BP in vivo, such as monocytes and macrophages,^(^ ^2^ ^)^ could be exposed to increased concentrations of the compound. In that case, immunological advert effects and increased risk of osteonecrosis of the jaw (ONJ)^(^ ^3^ ^)^ could became clinically relevant.

If we could add to the above mentioned concerns the need for daily subcutaneous injections, the prospects of the NE‐58024 compound becoming an improved version of the currently available BPs are less favorable.

## Disclosures

Michael Pazianas has nothing to disclose.
